# Earthquake in the time of COVID-19: The story from Croatia (CroVID-20)

**DOI:** 10.7189/jogh.10.010349

**Published:** 2020-06

**Authors:** Rok Čivljak, Alemka Markotić, Krunoslav Capak

**Affiliations:** 1 Dr Fran Mihaljević University Hospital for Infectious Diseases, Zagreb, Croatia; 2 University of Zagreb School of Medicine, Zagreb, Croatia; 3 Catholic University of Croatia, Zagreb, Croatia; 4 Faculty of Medicine of the University of Rijeka, Rijeka, Croatia; 5 Croatian Institute of Public Health, Zagreb, Croatia

On the hundredth day since days since the WHO was notified of the first cases of “pneumonia with unknown cause” in China, the COVID-19 pandemic, caused by SARS-CoV-2, has spread throughout the world to 195 countries with over 1.5 million cases and more than 85 000 deaths [1]. In early January, when we had just learned that a newly discovered disease was spreading in faraway China, only a few thought it would soon be coming to Croatia. Nevertheless, by February, when the epidemic was rapidly spreading in northern Italy, it was already quite likely that it would not bypass Croatia, and on 25 February 2020 the first case of COVID-19 was registered in our country [2].

The Government of the Republic of Croatia has established the National Civil Defense Headquarters in order to raise the level of preparedness of all the competent authorities, protect the health of Croatian citizens and coordinate all the services in the battle against SARS-CoV-2 in Croatia. The Deputy Prime Minister and Minister of the Interior was chosen to lead the headquarters, whose members are the Director of the Croatian Institute of Public Health, the Director of the Croatian Institute of Emergency Medicine, and the Director of the Dr Fran Mihaljević University Hospital for Infectious Diseases (UHID), who is also the President of the Croatian Society for Biosafety and Biosecurity. After the daily meetings, the headquarters issues press releases.

Together with other public services, all the health care institutions were quickly mobilized in preparation for the battle against the COVID-19 epidemic, with the UHID taking the leading role, from the establishment of quarantine to being declared the National Hospital for COVID-19. The Minister of Health issued the decision to proclaim the threat of a COVID-19 epidemic as an administrative measure, which does not indicate the degree of the threat but permits the Minister to mobilize all the resources in the health care system, redeploying ID physicians, other health care workers (HCWs) and equipment, in order to improve the administration of the health care system.

Unfortunately, what few had thought possible has come to pass. An infectious disease has transformed social and economic relations, as well as humanity as a whole, entering and altering every pore of society. Colleges and schools have closed their doors, students are attending classes from home, via television and computers, the majority of the public sector employees are working from home, while some, regrettably, have lost their jobs. The churches have also closed their doors.

At the end of the fourth week of the epidemic in Croatia, a total of 206 cases had been confirmed, with 49 newly diagnosed on 21 March 2020. When we thought that nothing worse could happen, on 22 March 2020, the citizens of Zagreb, the capital of Croatia, home to a quarter of the total Croatian population, were awakened at 6:24 am by an earthquake of 5.5 magnitude on the Richter scale, followed in the next 24 hours by 57 aftershocks. Due to extensive property damage, several hospitals had to be evacuated, including the UHID, where there were 86 patients at the moment, including 22 COVID-19 patients, 15 of whom were in the ICU. This additional misfortune has jeopardized the safety of us all, especially the most vulnerable members of our society: children, the elderly and the sick. A total of 27 persons were injured during the earthquake, of whom one, sadly a 15-year-old girl, died of her injuries.

We as HCWs were expected to remain clear-headed, gather all our strength, knowledge and skills, and provide our patients with the best of what was possible, and sometimes even the impossible. But we also remember the scene around our hospital buildings, where the old and young, staff and patients, corona-positive and negative, were scattered on the grass and the parking lot, shivering from the cold and fear.

Nevertheless, we survived that, too, and we will continue to survive, even when faced with greater challenges. We have demonstrated and proven that we cannot do without one another: physicians without nurses, patients without HCWs, HCWs without non-medical and support staff, parents without children, and children without parents.

**Figure:**
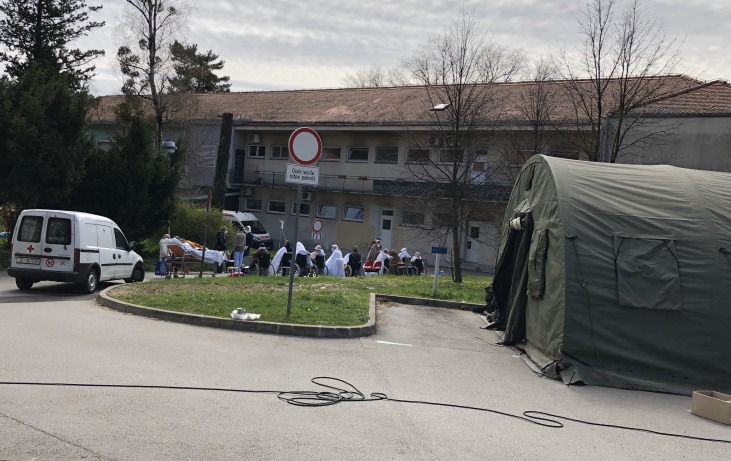
Photo: During the earthquake in Zagreb on 22 March 2020, 86 patients, including 22 COVID-19 patients, were evacuated from the extensively damaged hospital buildings (authors’ photo archive, used with permission).

In addition to property damage, we were quite apprehensive that the earthquake would accelerate the spread of the COVID-19 epidemic in Croatia since the earthquake, which left many homeless and fearful of earthquakes to come, triggered migrations to other parts of the country. However, six weeks since the first COVID-19 case and three weeks after the earthquake, only 1650 cases have been registered in Croatia (**Figure 1**), with 25 deaths (CFR of 1.5%) [3], making Croatia a country with one of the lowest rates of COVID-19 infection in Europe: 343 cases and 5 deaths per million inhabitants [4].

**Figure 1:**
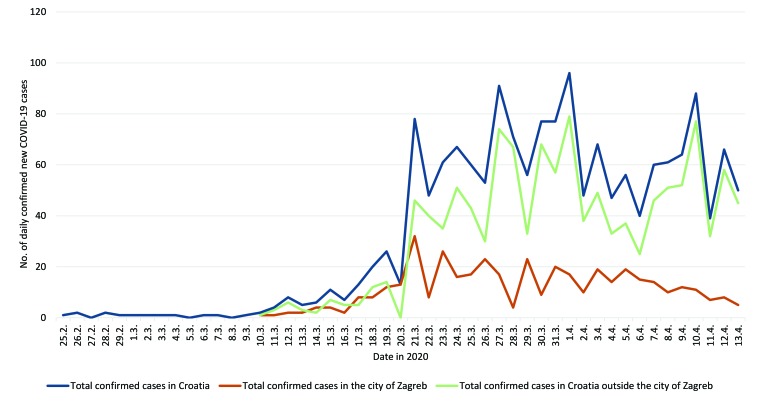
The number of confirmed COVID-19 cases in Croatia and the city of Zagreb six weeks after the first COVID-19 case and three weeks after the earthquake.

The subtitle of the previously cited article by Čivljak et al. [2], *What's next?,* warns of the looming danger from infectious diseases and the need for ongoing surveillance, because respiratory infections such as coronavirus diseases (SARS, MERS and COVID-19) are major threats to humanity. Nevertheless, no one expected that a second misfortune, the earthquake, would occur during this pandemic in our little country.

All HCWs, including physicians, nurses, technicians, laboratory personnel, caregivers and other staff who come into contact with patients, are being directly or indirectly exposed to the infected and sick, which places them at risk. At the UHID, among 691 employees, only two have acquired SARC-CoV-2 infection in the post-earthquake period: one nurse and one ancillary worker. On the national level, no increase in the incidence of COVID-19 was experienced in the post-earthquake period, as shown in **Figure 1**.

Moreover, owing to the chronic shortage of medical personnel throughout the entire health care system, particularly now when the demands are increasing, we are being expected to make great sacrifices in order to provide the best possible care to all citizens. An additional burden is that 216 HCWs in Croatia have COVID-19, while another 562 are in self-isolation at the moment. All of them are currently unable to perform their regular professional duties and the number may soon increase. The Minister of Health has stated that the health care system is not in jeopardy for now and the ministry is redeploying HCWs in order to fill the most crucial positions.

This third coronavirus epidemic underscores the need for the ongoing surveillance of infectious disease trends throughout the world. However, in combination with a natural disaster, such as earthquake, the risk for increasing the number of the infected, as well as outbreaks of other infectious diseases, is higher. As shown in the systematic review by Suk et al., cascading effects of post-disaster outbreaks are possible after earthquakes, such as outbreaks of *Salmonella*, chickenpox, or other infectious diseases in general [5].

By now, due to a well-organized public health system and coordinated outbreak response, Croatia has overcome the current challenges, including extensive damage to hospitals, shortage of hospital personnel, and disruption of supply chains. We hope that everything done so far will provide the basis for stopping this epidemic in Croatia and mitigating the damage done from the COVID-19 epidemic and the recent earthquake.

## References

[1] WHO. Coronavirus disease 2019 (COVID-19); situation report – 80. Available: https://www.who.int/docs/default-source/coronaviruse/situation-reports/20200409-sitrep-80-covid-19.pdf?sfvrsn=1b685d64_2. Accessed: 10 April 2020.

[2] Čivljak R Markotić A Kuzman I The third coronavirus epidemic in the third millennium: what’s next? Croat Med J. 2020;61:1-4. 10.3325/cmj.2020.61.1 32118371 PMC7063555

[3] HZJZ – Croatian Institute of Public Health. Koronavirus – najnoviji podatci. Available: https://www.hzjz.hr/priopcenja-mediji/koronavirus-najnoviji-podatci/. Accessed: 10 April 2020.

[4] Worldometer. Confirmed Cases and Deaths by Country, Territory, or Conveyance. Available: https://www.worldometers.info/coronavirus/. Accessed: 10 April 2020.

[5] Suk JE Vaughan EC Cook RG Semenza JC Natural disasters and infectious disease in Europe: a literature review to identify cascading risk pathways. Eur J Public Health. 2019; ckz111. Online ahead of print. 10.1093/eurpub/ckz111 31169886 PMC7536539

